# Spermatic Truss

**Published:** 1878-01-20

**Authors:** 


SPERMATIC TRUSS.—There may be seen in our advertising department a notice of a Spermatic Truss, manufactured by the Cooper Truss Company of Pittsburg, Pa.

As we have little faith in internal medication for obstinate and protracted cases of spermatorhoea, we were favorably struck with this appliance at first sight. We have examined a sample sent us and applied it to a subject.

Its action is mechanical; it is simple in construction, easy of application, not costly, can be worn with entire comfort to the patient, and so snugly adapts itself to the parts that erection can not occur, and emission is absolutely prevented.

There occurs to us another important application of this instrument which the proprietors seem strangely to have overlooked, and that is as a means of relieving obstinate cases of incontinence of urine in children.

